# Tumor-directed evolution of VSVΔ51M produces novel viruses with enhanced antitumor efficacy

**DOI:** 10.3389/fmolb.2026.1656006

**Published:** 2026-05-08

**Authors:** Omar A. Albaradie, Abdulaziz Molham Moglan, Yahya Marwan Samman, Fares Fayez Alsayegh, Hussam Mohsen Alharbi, Nada Almarghalani, Khulood Alotaibi, Mohammed M. Jalal, Malik A. Altayar, Nizar H. Saeedi, Hanan A. Niaz, Samer Zakri, Adnan Almuzaini, Feras Kaboha, Saleh Y. Alabbas, Yasmeen A. Albalawi, Abeer Alsofyani, Adrian Pelin, May Abdulaiz Alsayb, Shymaa Damfo, Ahmad Bakur Mahmoud, Almohanad A. Alkayyal

**Affiliations:** 1 College of Medicine, King Saud bin Abdulaziz University for Health Sciences, Jeddah, Saudi Arabia; 2 King Abdullah International Medical Research Center, Jeddah, Saudi Arabia; 3 College of Science, King Abdulaziz University, Jeddah, Saudi Arabia; 4 Department of Medical Laboratory Technology, Faculty of Applied Medical Sciences, University of Tabuk, Tabuk, Saudi Arabia; 5 King Saud bin Abdulaziz University for Health Sciences, Ministry of National Guard Health Affairs, Jeddah, Saudi Arabia; 6 Infectious Disease Research Department, King Abdullah International Medical Research Centre, Ministry of National Guard Health Affairs, Jeddah, Saudi Arabia; 7 Experimental Medicine Department, King Abdullah International Medical Research Centre, Jeddah, Saudi Arabia; 8 Immunology Research Program, King Abdullah International Medical Research Center, Jeddah, Saudi Arabia; 9 Department of Biology, College of Science, Jouf University, Sakaka, Aljouf, Saudi Arabia; 10 Department of Cellular Therapy and Cancer Research, King Abdullah International Medical Research Center, Jeddah, Saudi Arabia; 11 Department of Cellular and Molecular Pharmacology, University of California, San Francisco, San Francisco, CA, United States; 12 Gladstone Institute of Data Science and Biotechnology, J. David Gladstone Institutes, San Francisco, CA, United States; 13 College of Applied Medical Sciences, Taibah University, Almadinah Almunwarah, Saudi Arabia; 14 Health and Life Research Center, Taibah University, Almadinah Almunwarah, Saudi Arabia; 15 Department of Pharmacognosy and Pharmaceutical Chemistry, College of Pharmacy, Taibah University, Almadinah Almunwarah, Saudi Arabia; 16 Immunology Research Program, King Abdullah International Medical Research Center, Riyadh, Saudi Arabia

**Keywords:** oncolytic virotherapy, personalized therapy, vesicular stomatitis virus, viral evolution, VSVΔ51M

## Abstract

Oncolytic virotherapy has gained enormous attention and is undergoing extensive research, specifically, the Vesicular Stomatitis Virus (VSV). VSV has been shown to provide a potential tool on which many modifications can be introduced to improve its efficacy. Here we report utilizing a modified VSV with a deletion mutation at the 51 amino acid of its matrix protein (VSVΔ51), designed to leverage the innate antiviral immunity of healthy cells through the interferon pathway. We serially passaged VSVΔ51M thirty times in B16F10 and LLC1 cell lines. Plaque purification was performed post-passaging to ensure viral homogeneity. Using an ATP release-based assay, we demonstrated that serial passaging significantly enhanced the oncolytic activity of VSVΔ51M *in vitro* without increasing its toxicity in normal fibroblasts. Whole-genome sequencing of the resultant viruses detected several mutations in the viral glycoprotein and the large RNA polymerase: VSV-P30-LLC1 (VSV-G gene: N25T; VSV-L gene: S1538F) and VSV-P30-B16F10 (VSV-G gene: K2E, I53V, E369K). Most likely, these mutations are responsible for the enhanced efficacy observed both *in vitro* and *in vivo*. In silico studies further supported these findings, revealing an increased affinity of the VSV-P30-B16F10 glycoprotein for the LDLR receptor. Additionally, the therapeutic index of VSV-P30-B16F10 improved in the B16F10 peritoneal tumor model. Our findings suggest that serial passaging of VSVΔ51M generates novel variants with enhanced oncolytic profiles while maintaining oncoselectivity. However, further investigation, in preclinical and clinical settings, is necessary to validate the safety and efficacy of these novel variants.

## Introduction

Cancer is a worldwide leading cause of morbidity and mortality. As of 2022, it ranked the second most common cause of death in the United States (Siegel et al., 2022). In 2020, an estimated 19.3 million new cancer cases worldwide were recorded (Sung et al., 2021). According to GLOBOCAN 2020, it is estimated that by the year 2040, there will be 28.4 million cases of cancer, which is a 47% increase from 2020. However, this percentage could increase depending on the rising risk factors linked to globalization and an expanding economy (Siegel et al., 2022).

Despite the recent advances in antitumor therapy, in addition to the observed reduction in cancer mortality and morbidity, it remains a serious problem that necessitates novel therapeutic approaches (Siegel et al., 2022). Different treatment approaches have been developed and are being used in medical practice as a standard of care. Yet, most available treatments are either inefficacious and may result in intolerable adverse effects at higher doses or efficacious but highly invasive, to an extent where the burden of the treatment may become more than the disease itself (Arruebo et al., 2011). In the sense of providing a more efficacious treatment and better quality of life for cancer patients, novel treatment approaches have become a major preoccupation of modern cancer research.

One of the current promising cancer treatments undergoing extensive research is Oncolytic Virotherapy. Oncolytic viruses (OVs) have the ability to destroy cancer cells by two main mechanisms: (a) direct cell lysis through the invasion of cancerous cells and (b) induction of host immune reaction against the tumor (Goradel et al., 2021). When the virus initially enters a cancerous cell, it goes through the normal course of viral replication. By repeating this self-amplifying cycle, high volumes of viral particles can be reached *in situ*, eventually destroying the tumor while sparing the surrounding viable tissue. Since the natural evolution of cancer cells involves evading recognition by the immune system, tumors in theory, are more susceptible to infection by viruses as a result of cellular changes that lead to defective anti-viral pathways (Lemos de Matos et al., 2020).

In the hopes of discovering an optimum oncolytic viral strain, numerous viruses have been experimented with. One of the well-studied viruses in the field is Vesicular Stomatitis Virus (VSV). Vesicular Stomatitis Virus (VSV) is a particularly promising oncolytic platform due to several advantages: its rapid replication cycle, broad mammalian cell tropism mediated by the LDLR receptor, cytoplasmic replication (minimizing integration risk), and ease of genetic manipulation (Hastie and Grdzelishvili, 2012; Ge et al., 2010). VSV is generally non-pathogenic and rarely causes symptomatic disease in humans, resulting in low levels of preexisting immunity in the human population (Hastie and Grdzelishvili, 2012; Ge et al., 2010). Normal, healthy cells do not have a direct mechanism to evade or block VSV infection (Stojdl et al., 2003). Instead, they produce type-1 interferons (IFNs) that activate anti-viral immune responses (Stojdl et al., 2003). Many tumor cells exhibit defective or attenuated type-1 interferon (IFN) responses, rendering them more susceptible to viral infection compared to healthy cells (Lemos de Matos et al., 2020; Pearson et al., 1999).

Furthermore, a methionine deletion at position 51 of the matrix protein of VSV (VSVΔ51) has been shown to enhance innate antiviral immunity in healthy cells, improving the oncoselectivity profile of the virus (Hastie and Grdzelishvili, 2012; Stojdl et al., 2003). Infection of embryonic fibroblasts with either wild-type VSV (VSV-WT) or VSVΔ51M demonstrated that cell viability was preserved in VSVΔ51-infected cells compared to VSV-WT. Additionally, following *in vivo* injection of VSVΔ51M and VSV-WT, interferon-α was four-fold elevated in VSVΔ51-injected mice compared to VSV-WT (Stojdl et al., 2003). This attenuation, via a defective ability to dial down innate antiviral immunity in healthy cells, offers an enhanced oncoselective profile and a wider therapeutic index. Therefore, in this study, we hypothesize that serial passaging of VSVΔ51M on two cancer cell lines, B16F10 and LLC1, will generate novel oncolytic viruses that possess a superior oncotoxic profile, while preserving the original oncoselective mutation of VSVΔ51. Consequently, the generated viruses will be tested for their *in vitro* and *in vivo* safety and efficacy.

## Materials and methods

### Cells and viruses

The VSVΔ51M is a mutated Vesicular Stomatitis Virus developed in Alkayyal’s and Mohamoud’s labs as described elsewhere (Abdulal et al., 2023; Alkayyal et al., 2023). The murine cell lines B16F10 (ATCC CRL-6475) and LLC1 (ATCC CRL-1642) used in this study were obtained from Dr. Nada Zaidan and Dr. Khalid Shah (KACST- BWH Centre of Excellence for Biomedicine, Riyadh, Saudi Arabia). In addition, the VERO cell line (ATCC CCL-81) and the human lung cancer cell line, A549 (ATCC CCL-185), were kind gifts from Mr. Suhail Melibary (King Abdulaziz University Hospital, Jeddah, Saudi Arabia). All cell lines were maintained in Dulbecco’s modified Eagle’s medium (DMEM) supplemented with 10% fetal bovine serum (Sigma Aldrich, Saudi Arabia) and incubated in 5% CO_2_ at 37 °C. Cell lines with passages <10 were aliquoted and stored at −80 °C or Liquid nitrogen and used throughout the experiment. B16F10 (murine melanoma) and LLC1 (murine lung carcinoma) cell lines were chosen to represent diverse solid tumor types commonly used in oncolytic virus studies, allowing assessment of whether adaptations are tumor-type specific or broadly applicable.

### Viral passaging

**FIGURE 1:**
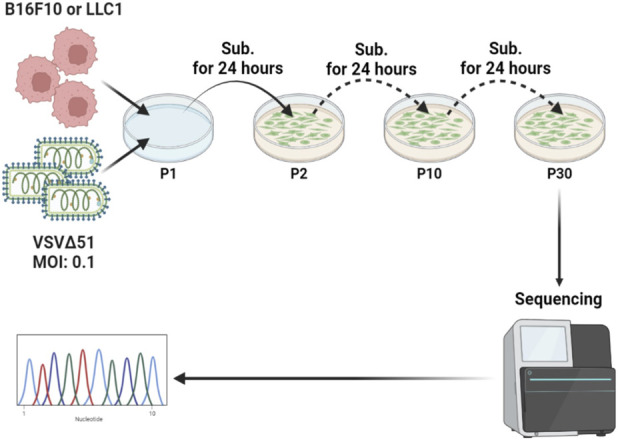
Scheme of viral passaging. VSVΔ51M was used to infect two cancer cell lines, B16F10 and LLC1, at an MOI of 0.1following Passage 1 (P1), was used to infect the next passage. The resultant four viruses after P10 and P30 were referred to as VSV-P10-LLC1, VSV-P10-B16F10, VSV-P30-LLC1, and VSV-P30-B16F10 throughout the study.

B16F10 and LLC1 were seeded into 6-cm plates having a confluency of ∼95% and incubated at 37 °C. Both had different infection periods, B16F10 being 24 h post-seeding while LLC1 is 48 h. After aspirating the media, cell monolayers were infected with an MOI of 0.1 in FBS-free DMEM and incubated for an hour, with a 15-min interval of rocking in between. Then, the media containing the unbound virus was aspirated and the fresh 10% cDMEM was added. The next day, the cytopathic effect of the infected plate was estimated under the light microscope; 3 mL were collected into three 1.5 mL Eppendorf tubes and stored in −80 °C. In addition, 100 µL of the supernatant with DMEM free of FBS was used to infect the subsequent passage. Passaged VSVΔ51M over B16F10 is referred to as VSV-P10-B16F10 (after 10 passages) or VSV-P30-B16F10 (after 30 passages). Similarly, VSVΔ51M passaged over LLC1 is referred to as VSV-P10-LLC1 or VSV-P30-LLC1. Figure 1 shows a scheme of the process of viral passaging.

### Viral titration

1 mL of cDMEM containing 4.5X10^5^ VERO cells was seeded into 12 well-plate and incubated for 24 h at 37 °C and 5% CO_2_. 5 μL was extracted from the stock and serially diluted in serum-free DMEM throughout the dilution 10^–2^ - 10^–10^. The media from the overnight seeded 12 well-plate was aspirated and infected with a volume of 0.3 mL of each virus dilution. Subsequently, the plate was incubated for 1 h. Post incubation time, the infectants were aspirated, and 1 mL of 3% agarose overly with 15% cDMEM, in a 2:1 ratio, was added to each well. After 1 day, each well was fixed with 1 mL of methanol-acetic acid fixative and incubated at room temperature for 24 h. Post-gel detachment, 1 mL of Coomassie Blue was added into each well. Conclusively, the concentration of the virus was calculated by taking the average of plaques in two consecutive wells.

### Viral plaque purification

The plaque purification step was performed to ensure the virus is homogeneous and free of various viral clones. 1X10^6^ VERO cells were seeded into a 6-well plate and incubated for 1 day. 5 μL was extracted from the heterogenous stock virus and serially diluted in serum-free DMEM throughout the dilutions 10^–2^ - 10^–10^. Each well is infected with an infection volume of 500 µL and incubated for 1 h. After the incubation, 2 mL of 3% agarose overly with 15%cDMEM, in a 2:1 ratio, was added to each well. Forty-eight hours post-infection, the largest visible plaque was marked and collected via a 200 µL pipet tip as it was inserted through the agarose and resuspended into a 1 mL serum-free DMEM. The collected viral suspension was expanded and used in further experiments.

### Viral cytotoxicity/cell viability

2X10^4^ of the desired cells were seeded into a 96-well plate and incubated for 24 h. After incubation, the cells were infected with the serially diluted virus having an MOI range of 0.0001–100 pfu/cell. Post-infection, the plate was incubated for 48 h before adding the CellTiterGlo-reagent to each well. To measure the amount of ATP present in each well, which is a representative readout of active cells, the luminescent Cell Viability assay reagent was used. The findings were quantified using an ELISA reader at a wavelength of 560 nm. All *in vitro* cytotoxicity experiments were performed in triplicate (n = 3 biological replicates).

### *In vivo* experiments

6x10^6^ B16f10 cells were collected and washed twice with PBS, to ensure that the cells are serum-free, and then centrifuged. Three Eppendorf tubes were prepared with 2x10^6^ B16F10 cells. The three tubes were infected *in vitro* with VSV-P30-B16F10 or VSVΔ51M at an MOI of 0.1 or phosphate-buffered saline (PBS) and incubated for 1 h. Subsequently, 100 µL of the infectant, containing 2 × 10^6^ B16f10 and 2 × 10^5^ pfu viral particles, was injected intraperitoneally (IP) into 6–9 weeks old female C57Bl/6 mice (5/group) bred by KAIMRC Animal Vivarium. Conversely, five C57Bl/6 mice were injected with 100 µL of B16F10 cells with PBS as a control group. The mice were monitored for survival every day by veterinarians at KAIMRC. The mice were managed and kept under veterinary oversight, following institutional protocols and in compliance with the guidelines set forth by the KAIMRC’s Institutional Animal Care and Use Committee (IACUC).

### RNA extraction, cDNA synthesis, and viral whole genome sequencing

The Purified viral suspension was RNA isolated via the QIAamp Viral RNA Mini kit (Qiagen) in compliance with the manufacturer’s instructions; the resultant RNA concentration was measured via a Nanodrop. Thereafter, the isolated RNA was converted into cDNA by the High-Capacity cDNA Reverse Transcription Kit (Qiagen) and finally stored at −80 °C until further processing.

For genomic analysis, whole genome sequencing (WGS) of the viral cDNA was performed using the Illumina MiSeq platform. Library preparation was carried out using standard protocols suitable for low-input RNA viral genomes. Sequencing reads were quality-checked, trimmed, and assembled using a combination of bioinformatics tools. The assembled viral genomes were aligned with the parental virus reference sequence using Geneious Prime (Biomatters) and CLC Genomics Workbench (Qiagen). Mutations, including single-nucleotide polymorphisms (SNPs) and insertions/deletions (indels), were identified through comparative genomic analysis to detect variations acquired during the viral passaging process.

### *In-silico* studies

The protein sequence of VSV was searched in the Protein Data Bank database (PDB) to determine corresponding protein crystal structures. Protein sequences were aligned with 5OYL as the wild-type sequence, which was retrieved from the PDB database (Nikolic et al., 2018). The Clustal Omega was used to generate the sequence alignment (Madeira et al., 2024). The structure of VSV was optimized for use in docking analysis by the QuickPrep tool of MOE (Molecular Operating Environment, Chemical Computing Group ULC, 2024; https://www.chemcomp.com/en/Products.htm). Missing atoms in the crystal structure, including hydrogen atoms, were corrected after determining the ionization state. Water molecules that are not involved in receptor-ligand interaction were deleted. The docking analysis was performed on the most stable conformation of the structure after energy minimization. The output structure after optimization was the structure for the docking and binding analysis. MOE protein contacts tool (ULC, 2024) was used to identify any interactions between mutated sequences and low-density lipoprotein receptor (LDLR). Protein-protein interactions were visualized as a contact list that included bound amino acids along with the energy values and frequencies. All structural insights and binding affinities reported herein are predicted based on *in silico* modeling and have not been experimentally validated.

### Statistical analysis

All statistical analyses were performed using GraphPad Prism version 9.0 (GraphPad Software, San Diego, CA). For *in vitro* data, statistical significance was assessed using the Student’s *t*-test. For *in vivo* experiments, Kaplan–Meier survival curves were generated, and differences in survival between groups were analyzed using the log-rank (Mantel–Cox) test. A *P*-value of <0.05 was considered statistically significant in all analyses.

## Results

### Experimental passaging of VSVΔ51M produced a trend of enhanced oncotoxicity following ten passages over B16F10 but not over LLC1

**FIGURE 2:**
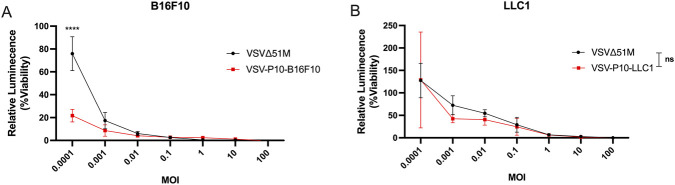
**(A)** B16F10 murine melanoma cells and **(B)** LLC1 murine lung carcinoma cells were infected with either parental VSVΔ51M or VSV-P10 variants (VSV-P10-B16F10 or VSV-P10-LLC1) at a range of multiplicities of infection (MOIs; 100 to 0.0001). Cell viability was assessed 48 h post-infection using a luminescence-based ATP assay. Statistical significance was determined by unpaired two-tailed Student’s t-test at each MOI. ****P < 0.0005; ns = not significant.

To investigate whether early-stage adaptation influences oncolytic potency, we assessed the cytotoxic effects of VSVΔ51M following ten passages on B16F10 and LLC1 cells. Viral cytotoxicity was evaluated using a luminescent ATP-based viability assay after 48 h of infection at varying multiplicities of infection (MOIs), ranging from 0.0001 to 100. In the B16F10 murine melanoma cell line (Figure 2A), VSV-P10-B16F10 exhibited significantly greater cytotoxicity compared to the parental VSVΔ51M virus, but only at the lowest MOI tested (0.0001), where viability dropped by more than 50% relative to VSVΔ51M (P < 0.0005). No significant differences were observed at higher MOIs, indicating that early passaging begins to enhance viral potency but is most evident under low-dose conditions, which may reflect more sensitive measures of viral fitness. In contrast, in the LLC1 murine lung carcinoma cell line (Figure 2B), VSV-P10-LLC1 did not show any significant improvement in cytotoxicity compared to the parental virus at any MOIs tested. Despite a slight downward trend in viability at lower MOIs, the differences were not statistically significant, and variability remained high across replicates. These findings suggest that early-stage adaptive mutations may begin to enhance oncolytic potency in a cell line-specific manner, with more pronounced effects in B16F10 than in LLC1. The data also support the need for continued passaging to achieve stronger and broader enhancements in viral efficacy.

### VSV-P30-B16F10 exhibits potent oncotoxicity and maintains oncoselectivity

**FIGURE 3:**
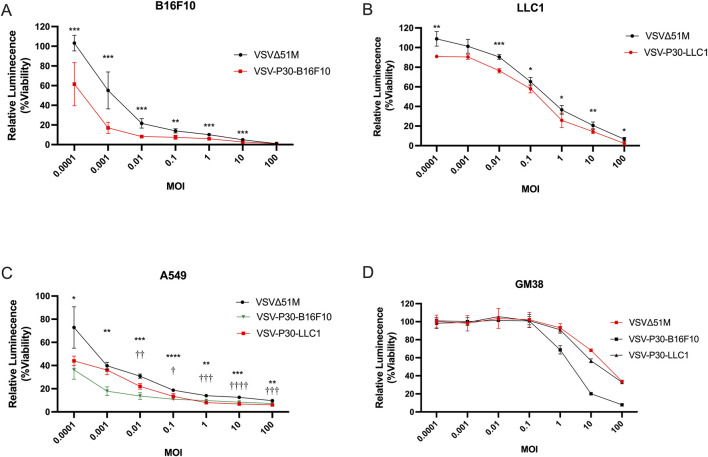
**(A)** B16F10 murine melanoma cells, **(B)** LLC1 murine lung cancer cells **(C)** A549 human lung carcinoma cells, and **(D)** GM38 normal human fibroblasts were infected with parental VSVΔ51, VSV-P30-B16F10, or VSV-P30-LLC1 at a range of multiplicities of infection (MOIs; 0.0001–100). Cell viability was assessed 48 h post-infection using the CellTiter-Glo luminescence assay. Statistical significance for the B16F10 **(A)** and LLC **(B)** panels was determined using an unpaired two-tailed Student’s *t*-test, while significance for the A549 **(C)** panel was assessed using two-way ANOVA followed by multiple unpaired *t*-tests with false discovery rate (FDR) correction for multiple comparisons. Asterisks (*) denote comparisons between VSVΔ51M and VSV-P30-B16F10, and daggers (†) denote comparisons between VSVΔ51M and VSV-P30-LLC1. The levels of significance are indicated as follows: P < 0.05 (* or †), P < 0.01 (** or ††), P < 0.001 (*** or †††), and P < 0.0001 (**** or ††††).

To further assess the viral potency and selectivity after 30 passages, we evaluated the cytotoxicity of VSV-P30-B16F10 and VSV-P30-LLC1 across both murine and human cancer cell lines, as well as normal fibroblasts. In the murine melanoma B16F10 cell line and LLC1 murine lung cancer cell line, VSV-P30-B16F10 and VSV-P30-LLC1 demonstrated significantly enhanced cytotoxicity, respectively, compared to the parental VSVΔ51M across a broad range of MOIs (0.0001–10). The improvement in cytotoxicity was most pronounced at low MOIs, with viability reduced by over 80% at MOI 0.001 (*P* < 0.005). These findings suggest that extended passaging in B16F10 and LLC1 cells might drive the acquisition of adaptive mutations that improve oncolytic activities in a cell line-specific manner (Figures 3A,B).

We subsequently assessed both VSV-P30 variants in the human lung carcinoma A549 cell line. VSV-P30-B16F10 (*) exhibited consistently greater cytotoxicity compared to VSVΔ51M across all tested MOIs (*P < 0.05 to ****P < 0.0001). In contrast, VSV-P30-LLC1 (†) displayed significant cytotoxic improvements relative to the parental virus only at intermediate and higher MOIs (≥0.01). Pairwise comparisons indicated that VSV-P30-B16F10 consistently outperformed VSV-P30-LLC1, suggesting that B16F10-derived mutations conferred superior functional adaptation with potentially broader tumor tropism (Figure 3C). To evaluate oncoselectivity, the cytotoxicity was assessed in GM38 human dermal fibroblasts. All viral variants exhibited minimal cytotoxicity at MOIs ≤1, with no significant deviation from baseline viability. At higher MOIs (10 and 100), VSV-P30-B16F10 induced moderate cytotoxicity compared to the parental virus, suggesting a slight compromise in selectivity at supraphysiological doses, which supports its retained safety profile (Figure 3D).

These results collectively demonstrate that serial passaging of VSVΔ51M in B16F10 cells enhances oncolytic potency while preserving selectivity to a certain extent, and may confer cross-species efficacy in human cancer models.

### VSV-P30-B16F10 shows enhanced oncolytic activity *in vivo*

**FIGURE 4:**
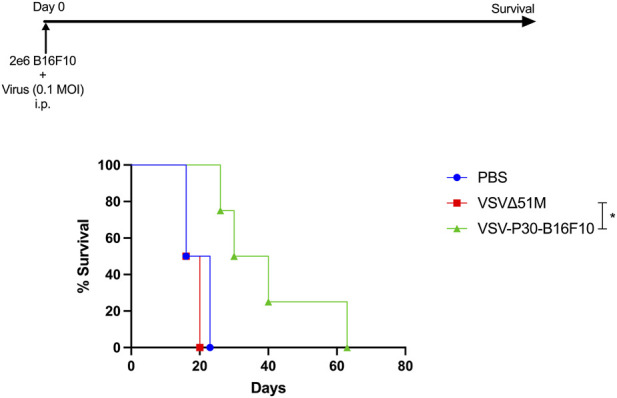
C57BL/6 mice (6–8 weeks old) were injected intraperitoneally (i.p.) with 2 × 10^6^ B16F10 melanoma cells that had been pre-incubated for 1 h at 37 °C with either VSVΔ51M, VSV-P30-B16F10 (MOI = 0.1; 5 × 10^5^ PFU), or PBS as a negative control. Tumor progression and overall survival were monitored over time. Mice treated with VSV-P30-B16F10–infected cells exhibited significantly prolonged survival compared to both the VSVΔ51M and PBS control groups. The median survival of the VSV-P30-B16F10 group was 35 days, compared to 18 and 19.5 days in the VSVΔ51M and PBS groups, respectively. Data represent a single experiment with n = 5 mice per group. Statistical significance was assessed using the Log-rank (Mantel–Cox) test (χ^2^ = 7.862, *P* = 0.0196), the Log-rank test for trend (χ^2^ = 5.396, *P* = 0.0202), and the Gehan–Breslow–Wilcoxon test (χ^2^ = 6.679, *P* = 0.0355). All analyses confirmed significant differences in survival curves (*P* < 0.05).

To evaluate therapeutic efficacy *in vivo*, VSV-P30-B16F10 was tested in a syngeneic murine B16F10 peritoneal tumor model in C57BL/6 mice (Figure 4). B16F10 cells (2 × 10^6^) were pre-infected with either VSV-P30-B16F10 or the parental VSVΔ51M strain at an MOI of 0.1 (equivalent to 5 × 10^5^ PFU) for 1 h at 37 °C, followed by intraperitoneal (i.p.) injection into 6–8-week-old C57BL/6 mice.

Mice receiving VSV-P30-B16F10–infected tumor cells exhibited significantly prolonged survival, with a median survival of 35 days, compared to 18 days in the VSVΔ51M group and 19.5 days in the PBS-treated control group (*P < 0.05*, log-rank test; Figure 4). Notably, VSV-P30-B16F10 extended survival in all treated animals, with some surviving for more than 50 days indicating a robust anti-tumor response. In contrast, all mice in the control and parental virus groups succumbed by day 21.

These results confirm that serial passaging of VSVΔ51M in B16F10 cells yields an adapted virus with superior *in vivo* efficacy, supporting its potential as a next-generation oncolytic virus.

### Identification of novel mutations in VSVΔ51M following serial passaging over B16F10 and LLC1

Serial passaging of VSVΔ51M over B16F10 and LLC1 cell lines led to the emergence of novel genetic mutations. We hypothesized that continuous passaging would drive the selection of adaptive mutations, potentially enhancing the virus’s oncolytic potency. To test this hypothesis, whole genome sequencing (WGS) was performed on two passaged viral isolates: VSV-P30-B16F10 and VSV-P30-LLC1.

WGS analysis revealed three missense mutations in VSV-P30-B16F10 and two missense mutations in VSV-P30-LLC1. Notably, all three mutations identified in VSV-P30-B16F10 were located within the VSV glycoprotein (VSV-G) gene: K2E, I53V, and E369K. In contrast, the mutations found in VSV-P30-LLC1 were distributed across two genes: S1538F in the VSV large polymerase (VSV-L) gene and N25T in the VSV-G gene. These findings suggest distinct adaptive pathways during viral evolution in different tumor microenvironments. A summary of the detected mutations is provided in Table 1. The VSV glycoprotein (G) mediates viral entry by binding to the LDLR receptor, while the large polymerase (L) protein is essential for viral RNA synthesis.

**TABLE 1:** Novel genetic mutations in VSVΔ51M have been detected following viral passaging.

Virus	Gene	Position	Codon change (Original/Novel)	Mutation (Oringinal/Novel)
VSV-P30-B16F10	G	2	AAG/GAG	K/E
	G	53	ATA/GTA	I/V
	G	369	GAA/AAA	E/K
VSV-P30-LLC1	G	25	AAC/ACC	N/T
	L	1,538	TCT/TTT	S/F

Viral whole genome sequencing (WGS) was performed to identify mutations that emerged following 30 serial passages of VSVΔ51M on B16F10 or LLC1 cancer cells. Viral RNA, was extracted from purified viral stocks, converted into cDNA, and subjected to high-throughput sequencing. The table summarizes non-synonymous mutations detected in the glycoprotein (G) and polymerase (L) genes of the passaged viruses, including codon-level changes and resulting amino acid substitutions. Mutations unique to VSV-P30-B16F10 or VSV-P30-LLC1 are listed by gene, nucleotide position, and codon/amino acid conversion.

### Structural insights predict enhanced binding affinity of the B16F10 variant toward LDLR

**FIGURE 5:**
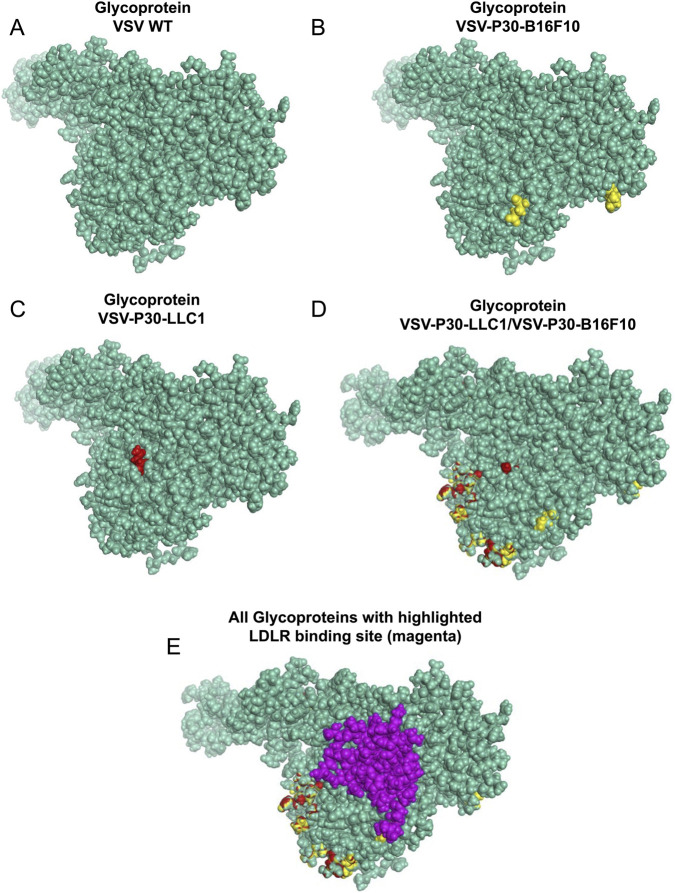
Structural comparison of VSV glycoproteins from wild-type and passaged variants, and their interaction with LDLR. **(A)** Surface representation of the wild-type VSVΔ51M glycoprotein (VSV WT) showing the unmutated structure. **(B)** Surface model of the VSV-P30-B16F10 glycoprotein, with mutation sites highlighted in yellow. **(C)** Surface model of the VSV-P30-LLC1 glycoprotein, with the mutation site highlighted in red. **(D)** Overlay of VSV-P30-B16F10 (yellow) and VSV-P30-LLC1 (red) variants mapped onto the glycoprotein surface, illustrating the spatial distribution of mutations. **(E)** Structural model showing the glycoproteins in complex with the LDL receptor (LDLR, shown in magenta), highlighting the proximity of the mutated residues (yellow and red) to the predicted LDLR binding interface. These structural differences may underlie the enhanced binding affinity and altered receptor interactions observed in mutant viruses.

Comparative analysis suggests that the VSV-P30-B16F10 viral sequence exhibits a higher binding affinity to the LDLR compared to both the VSVΔ51M and the VSV-P30-LLC1 variant. Multiple sequence alignment revealed both conserved and variable amino acid residues among the VSVΔ51, VSV-P30-B16F10, and VSV-P30-LLC1 viral sequences, as illustrated in (Supplementary Figure S1). An overview of the crystal structures for VSVΔ51, VSV-P30-B16F10, and VSV-P30-LLC1, along with key differences of residues, is presented in (Figure 5). Structural insight into VSV glycoproteins predicts that VSV-P30-B16F10 harbored two distinct surface-exposed mutations, whereas VSV-P30-LLC1 contained a single mutation as shown (Figures 5A–C). Overlay analysis highlighted that the mutations in VSV-P30-B16F10 and VSV-P30-LLC1 occupied different spatial locations on the glycoprotein surface (Figure 5D). Importantly, when modeled in complex with LDLR, the mutations in VSV-P30-B16F10 are predicted to cluster near the predicted receptor binding interface, while mutations in VSV-P30-LLC1 were more peripherally located (Figure 5E). These structural differences may contribute to the predicted enhanced LDLR binding affinity observed for the VSV-P30-B16F10 variant.

**FIGURE 6:**
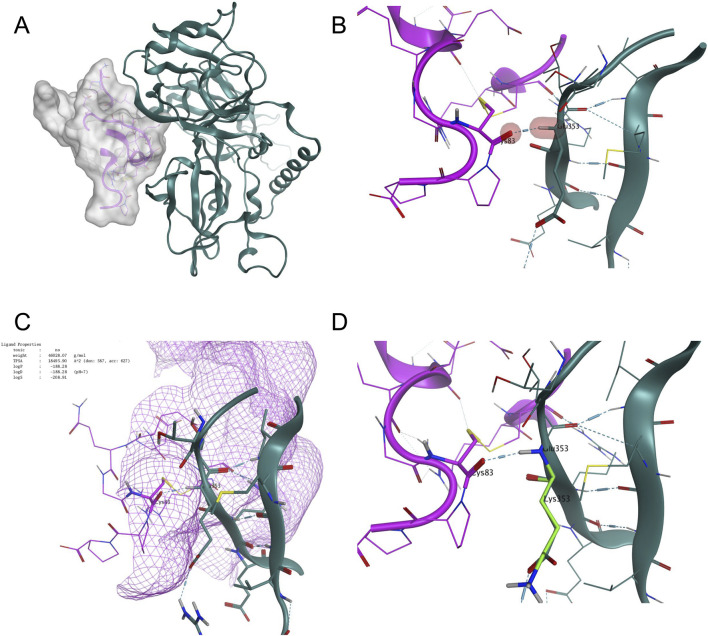
Structural modeling reveals enhanced interaction between VSV-P30-B16F10 and LDLR mediated by the E353K mutation. **(A)** Overall structure of the VSV glycoprotein (green) in complex with the LDL receptor (LDLR; magenta), shown in ribbon representation with the LDLR surface displayed in transparent grey. **(B)** Close-up view of the wild-type VSV-LDLR interaction highlighting residue Glu353, which forms a hydrogen bond with Cys83 of LDLR (residues indicated in red shading). **(C)** Surface mesh visualization of the binding interface, further illustrating the proximity of the interacting residues. **(D)** Structural model of the VSV-P30-B16F10 variant showing the Glu353 to Lys353 substitution (light green), which maintains the hydrogen bond with LDLR at Cys83 and is predicted to enhance binding affinity. Hydrogen bonds are indicated with dashed lines.

To further assess the binding affinity of the VSV-P30-B16F10 variant to LDLR, molecular docking was performed followed by peptide analysis. The resulting binding energy predicted that the VSV-P30-B16F10 variant binds to LDLR with greater affinity than the WT sequence, and significantly higher than the VSV-P30-LLC1 variant, as summarized in Table 2. Peptide analysis of the VSV/LDLR complex revealed stabilizing interactions, including hydrogen bonding between the viral sequence and LDLR (Figures 6A,B). In the VSVΔ51M structure, Glu353 forms a hydrogen bond with Cys83 on the LDLR (Figure 6C). However, in the VSV-P30-B16F10 variant, Glu353 is mutated to Lys353, a change predicted to enhance the electrostatic interaction with LDLR, thereby contributing to the increased binding affinity (Figure 6D; Table 3). This mutation likely plays a pivotal role in the improved viral targeting of LDLR-expressing tumor cells. Experimental validation of this predicted enhanced LDLR binding conferred by the E369K mutation could be achieved through further investigations including surface plasmon resonance and pseudovirus entry assays.

**TABLE 2:** VSV-P30-B16F10 exhibits higher predicted binding affinity to LDLR compared to VSV-P30-LLC1 and parental virus.

Ligand	Docking score
Parent (WT) VSVΔ51M	−74.6
VSV-P30-B16F10	−82.0
VSV-P30-LLC1	−64.1

**TABLE 3:** Effect of the E353K mutation on predicted binding affinity to LDLR.

Residue	D affinity
E353	0.0000
E353K	−0.6866

Molecular docking and interaction analysis were performed using the QuickPrep tool in MOE software to evaluate the binding affinity of VSV glycoprotein variants to the low-density lipoprotein receptor (LDLR). Docking scores were calculated for the parental virus (WT), VSV-P30-B16F10, and VSV-P30-LLC1 glycoproteins.

Binding affinity differences between the wild-type residue (E353) and the mutant residue (E353K) were assessed. The E353K substitution resulted in an increase in predicted binding affinity toward LDLR, with a ΔAffinity score of −0.6866 relative to the wild-type interaction. This enhanced interaction is predicted to contribute to the improved binding and oncotropism observed with the VSV-P30-B16F10 variant.

## Discussion

In this study, we investigated whether serial passaging of a modified Vesicular Stomatitis Virus (VSVΔ51) engineered with a methionine deletion at position 51 of its matrix (M) protein could enhance its oncolytic activity. Our findings demonstrate that viral passaging over murine cancer cell lines, particularly B16F10 melanoma cells, substantially improved the viral oncotoxic profile while preserving oncoselectivity.

Enhanced cytotoxicity of the adapted virus, VSV-P30-B16F10, was not only observed in its host cell line but also reproduced in a human lung carcinoma cell line (A549), suggesting that beneficial mutations acquired during viral evolution in murine cancer cells may be functionally translatable to human tumor contexts. Importantly, cytotoxicity remained low in GM-38 fibroblasts, indicating that selectivity for transformed cells was maintained.

Whole-genome sequencing of VSV-P30-B16F10 revealed three novel missense mutations in the glycoprotein (G) gene, which likely underlie the enhanced oncolytic phenotype. Structural modeling and docking analysis indicated that these mutations, particularly the Glu353 to Lys353 substitution, may strengthen binding affinity to LDLR, a known cellular entry receptor for VSV viruses (Nikolic et al., 2018). This improved interaction could contribute to more efficient viral entry and propagation in cancer cells.

Interestingly, the observed efficacy of VSV-P30-LLC1 was modest, perhaps underscoring the influence of intracellular cancer cell environment and cell-type-specific factors on viral evolution. However, the comparable oncolytic effects of VSV-P30-B16F10 and VSV-P30-LLC1 in A549 cells suggest that some beneficial mutations may confer broad-spectrum activity across multiple types of tumors. The more modest improvements observed with VSV-P30-LLC1 may reflect cell-intrinsic differences in antiviral defenses or slower accumulation of beneficial mutations in the LLC1 intracellular cancer cell environment compared to B16F10.

The results align with previous work by Seegers *et al.*, who demonstrated that serial passaging of a recombinant VSVΔ51M expressing p53 in resistant pancreatic cancer cells enhanced viral replication and efficacy through mutations in the G gene (Seegers et al., 2020). Similarly, our study supports the idea that adaptive evolution can refine viral tropism and potency without compromising safety.

Serial passaging has been successfully applied to other oncolytic viruses to enhance tumor-specific replication and efficacy. For example, Coxsackievirus CVB3 variant PD-H was adapted rapidly (within 10 passages) to the refractory colorectal carcinoma cell line Colo320 perhaps due to its smaller genome and higher mutation rate, whereas VSV, with its larger negative-sense RNA genome, required 30 passages in our study to achieve significant improvements (Elsner et al., 2024). These differences likely reflect variations in replication fidelity, viral genome size, and perhaps the host-cell interactions across different viral families. Similar adaptive evolution of oncolytic viruses has been observed in experimental and preclinical models, where directed evolution and selection strategies yield variants with enhanced tumor specificity and therapeutic properties. These approaches exploit the natural capacity of viruses to replicate, mutate, and adapt under selective pressure, supporting the concept that viral adaptation can contribute to improved antitumor activity in clinically relevant systems (Ronsard et al., 2014; Ronsard et al., 2015; Ronsard et al., 2017a; Raja et al., 2017; Ronsard et al., 2017b; Ronsard et al., 2019; Zainutdinov et al., 2019).

Numerous studies have explored the genetic engineering of VSV for improved oncolytic performance. Examples include the incorporation of cytokines (e.g., IFN-β), tumor-associated antigens, or alternative viral glycoproteins. For instance, VSV expressing the SARS-CoV-2 spike RBD has shown enhanced efficacy in melanoma models, and VSV-IFNβ has demonstrated remarkable tumor clearance in preclinical studies (Alkayyal et al., 2023; Moglan et al., 2023; Naik et al., 2012; Patel et al., 2015). Our findings offer a complementary strategy using adaptive passaging rather than exogenous gene insertion to enhance VSV’s therapeutic profile.

Nonetheless, transitioning oncolytic viruses into clinical use remains challenging. Systemic delivery is often hindered by pre-existing or treatment-induced antiviral immunity, limiting viral access to tumors (Shin et al., 2021). Approaches such as PEGylation, transient immunosuppression, or nanoparticle encapsulation have shown promise in mitigating this issue but may also dampen the immune-mediated component of viral therapy (Tesfay et al., 2013; Peng et al., 2013; Almstatter et al., 2015; Mendez et al., 2014; Tresilwised et al., 2012). Additionally, the immunosuppressive tumor microenvironment, driven by glycolysis (the Warburg effect) and lactate accumulation, can inhibit effector immune cells, thereby compromising therapeutic outcomes (Vander Heiden et al., 2009; Sukumar et al., 2015). Combinatorial regimens with metabolic modulators, such as dichloroacetate (DCA), may help reverse this suppression and improve the effectiveness of virotherapy (Meng et al., 2020).

A key limitation of the current *in vivo* model is the mode of viral delivery, as B16F10 tumor cells were pre-infected *ex vivo* prior to intraperitoneal injection, which does not fully recapitulate clinically relevant intratumoral or systemic administration. Future studies should further evaluate direct viral delivery routes to better model therapeutic applications. In addition, only female C57BL/6 mice were used, which may influence immune-mediated antitumor effects, as females typically mount stronger innate and adaptive immune responses than males; accordingly, inclusion of both sexes will be important to assess potential sex-dependent differences in efficacy and immune engagement (Klein and Flanagan, 2016), (Furman et al., 2014), (Fink and Klein, 2018). Serial passaging may also introduce unwanted mutations that perhaps could lead to off-target tropism or safety aspects, underscoring the need for comprehensive preclinical evaluation. Although viral replication was not directly quantified in this study, the enhanced cytotoxicity observed *in vitro* and improved survival *in vivo* strongly suggest increased replication fitness in tumor cells, which warrants further direct assessments in future studeis. Finally, while efficacy in the human A549 cell line suggests broader translational potential beyond the murine passaging models, additional tumor types must be tested to distinguish generalizable adaptations from cell line–specific effects.

## Conclusion

This study demonstrates that serial passaging of VSVΔ51M over murine cancer cell lines drives the evolution of variants with enhanced oncotoxic properties while preserving oncoselectivity. The emergence of novel mutations in the viral glycoprotein, particularly those that improve LDLR binding, appears to underlie the enhanced efficacy observed both *in vitro* and *in vivo*. These findings support the utility of experimental evolution as a viable strategy to optimize oncolytic viruses for cancer therapy. Future work should include preclinical evaluation of direct viral delivery, broader testing across diverse tumor models, and reverse-engineering of individual mutations to dissect their contributions. Collectively, these insights may accelerate the development of passaged VSV variants toward clinical translation.

## Data Availability

The complete genome sequences generated in this study are publicly available in the GenBank repository (NCBI) under accession numbers PZ289841 for VSVΔ51M-P30-B16F10 and PZ289968 for VSVΔ51M-P30-LLC1.

## References

[B1] Abdulal R. H. Malki J. S. Ghazal E. Alsaieedi A. A. Almahboub S. A. Khan M. Y. (2023). Construction of VSVDelta51M oncolytic virus expressing human interleukin-12. Front. Mol. Biosci. 10, 1190669. 10.3389/fmolb.2023.1190669 37255540 PMC10225647

[B2] Alkayyal A. A. Ajina R. Cacciabue M. Alkayyal A. A. Saeedi N. H. Hussain Alshehry T. (2023). SARS-CoV-2 RBD protein enhances the oncolytic activity of the vesicular stomatitis virus. Front. Immunol. 14, 1082191. 10.3389/fimmu.2023.1082191 36798114 PMC9927213

[B3] Almstatter I. Mykhaylyk O. Settles M. Altomonte J. Aichler M. Walch A. (2015). Characterization of magnetic viral complexes for targeted delivery in oncology. Theranostics 5 (7), 667–685. 10.7150/thno.10438 25897333 PMC4402492

[B4] Arruebo M. Vilaboa N. Saez-Gutierrez B. Lambea J. Tres A. Valladares M. (2011). Assessment of the evolution of cancer treatment therapies. Cancers (Basel) 3 (3), 3279–3330. 10.3390/cancers3033279 24212956 PMC3759197

[B5] Elsner L. Heimann L. Geisler A. Dieringer B. Knoch K. P. Hinze L. (2024). Fast track adaptation of oncolytic coxsackie B3 virus to resistant colorectal cancer cells - a method to personalize virotherapy. Biol. Proced. Online 26 (1), 11. 10.1186/s12575-024-00237-2 38664647 PMC11044309

[B6] Fink A. L. Klein S. L. (2018). The evolution of greater humoral immunity in females than males: implications for vaccine efficacy. Curr. Opin. Physiol. 6, 16–20. 10.1016/j.cophys.2018.03.010 30320243 PMC6181235

[B7] Furman D. Hejblum B. P. Simon N. Jojic V. Dekker C. L. Thiebaut R. (2014). Systems analysis of sex differences reveals an immunosuppressive role for testosterone in the response to influenza vaccination. Proc. Natl. Acad. Sci. U. S. A. 111 (2), 869–874. 10.1073/pnas.1321060111 24367114 PMC3896147

[B8] Ge P. Tsao J. Schein S. Green T. J. Luo M. Zhou Z. H. (2010). Cryo-EM model of the bullet-shaped vesicular stomatitis virus. Science 327 (5966), 689–693. 10.1126/science.1181766 20133572 PMC2892700

[B9] Goradel N. H. Baker A. T. Arashkia A. Ebrahimi N. Ghorghanlu S. Negahdari B. (2021). Oncolytic virotherapy: challenges and solutions. Curr. Probl. Cancer 45 (1), 100639. 10.1016/j.currproblcancer.2020.100639 32828575

[B10] Hastie E. Grdzelishvili V. Z. (2012). Vesicular stomatitis virus as a flexible platform for oncolytic virotherapy against cancer. J. Gen. Virol. 93 (12), 2529–2545. 10.1099/vir.0.046672-0 23052398 PMC4091291

[B11] Klein S. L. Flanagan K. L. (2016). Sex differences in immune responses. Nat. Rev. Immunol. 16 (10), 626–638. 10.1038/nri.2016.90 27546235

[B12] Lemos de Matos A. Franco L. S. McFadden G. (2020). Oncolytic viruses and the immune system: the dynamic duo. Mol. Ther. Methods Clin. Dev. 17, 349–358. 10.1016/j.omtm.2020.01.001 32071927 PMC7015832

[B13] Madeira F. Madhusoodanan N. Lee J. Eusebi A. Niewielska A. Tivey A. R. N. (2024). The EMBL-EBI job dispatcher sequence analysis tools framework in 2024. Nucleic Acids Res. 52 (W1), W521–W525. 10.1093/nar/gkae241 38597606 PMC11223882

[B14] Mendez N. Herrera V. Zhang L. Hedjran F. Feuer R. Blair S. L. (2014). Encapsulation of adenovirus serotype 5 in anionic lecithin liposomes using a bead-based immunoprecipitation technique enhances transfection efficiency. Biomaterials 35 (35), 9554–9561. 10.1016/j.biomaterials.2014.08.010 25154663 PMC4157089

[B15] Meng G. Li B. Chen A. Zheng M. Xu T. Zhang H. (2020). Targeting aerobic glycolysis by dichloroacetate improves Newcastle disease virus-mediated viro-immunotherapy in hepatocellular carcinoma. Br. J. Cancer 122 (1), 111–120. 10.1038/s41416-019-0639-7 31819179 PMC6964686

[B16] Moglan A. M. Albaradie O. A. Alsayegh F. F. Alharbi H. M. Samman Y. M. Jalal M. M. (2023). Preclinical efficacy of oncolytic VSV-IFNbeta in treating cancer: a systematic review. Front. Immunol. 14, 1085940. 10.3389/fimmu.2023.1085940 37063914 PMC10104167

[B17] Naik S. Nace R. Barber G. N. Russell S. J. (2012). Potent systemic therapy of multiple myeloma utilizing oncolytic vesicular stomatitis virus coding for interferon-beta. Cancer Gene Ther. 19 (7), 443–450. 10.1038/cgt.2012.14 22522623 PMC3380174

[B18] Nikolic J. Belot L. Raux H. Legrand P. Gaudin Y. A A. A. (2018). Structural basis for the recognition of LDL-receptor family members by VSV glycoprotein. Nat. Commun. 9 (1), 1029. 10.1038/s41467-018-03432-4 29531262 PMC5847621

[B19] Patel M. R. Jacobson B. A. Ji Y. Drees J. Tang S. Xiong K. (2015). Vesicular stomatitis virus expressing interferon-beta is oncolytic and promotes antitumor immune responses in a syngeneic murine model of non-small cell lung cancer. Oncotarget 6 (32), 33165–33177. 10.18632/oncotarget.5320 26431376 PMC4741756

[B20] Pearson A. S. Koch P. E. Atkinson N. Xiong M. Finberg R. W. Roth J. A. (1999). Factors limiting adenovirus-mediated gene transfer into human lung and pancreatic cancer cell lines. Clin. Cancer Res. 5 (12), 4208–4213. 10632362

[B21] Peng K. W. Myers R. Greenslade A. Mader E. Greiner S. Federspiel M. J. (2013). Using clinically approved cyclophosphamide regimens to control the humoral immune response to oncolytic viruses. Gene Ther. 20 (3), 255–261. 10.1038/gt.2012.31 22476202 PMC3806053

[B22] Raja R. Ronsard L. Lata S. Trivedi S. Banerjea A. C. (2017). HIV-1 Tat potently stabilises Mdm2 and enhances viral replication. Biochem. J. 474 (14), 2449–2464. 10.1042/BCJ20160825 28468838 PMC5509382

[B23] Ronsard L. Lata S. Singh J. Ramachandran V. G. Das S. Banerjea A. C. (2014). Molecular and genetic characterization of natural HIV-1 Tat Exon-1 variants from North India and their functional implications. PLoS One 9 (1), e85452. 10.1371/journal.pone.0085452 24465566 PMC3900424

[B24] Ronsard L. Raja R. Panwar V. Saini S. Mohankumar K. Sridharan S. (2015). Genetic and functional characterization of HIV-1 Vif on APOBEC3G degradation: first report of emergence of B/C recombinants from North India. Sci. Rep. 5, 15438. 10.1038/srep15438 26494109 PMC4616021

[B25] Ronsard L. Ganguli N. Singh V. K. Mohankumar K. Rai T. Sridharan S. (2017a). Impact of genetic variations in HIV-1 tat on LTR-mediated transcription *via* TAR RNA interaction. Front. Microbiol. 8, 706. 10.3389/fmicb.2017.00706 28484443 PMC5399533

[B26] Ronsard L. Rai T. Rai D. Ramachandran V. G. Banerjea A. C. (2017b). *In silico* analyses of subtype specific HIV-1 Tat-TAR RNA interaction reveals the structural determinants for viral activity. Front. Microbiol. 8, 1467. 10.3389/fmicb.2017.01467 28848502 PMC5550727

[B27] Ronsard L. Yousif A. S. Ramesh J. Sumi N. Gorman M. Ramachandran V. G. (2019). *In-Vitro* subtype-specific modulation of HIV-1 trans-activator of transcription (Tat) on RNAi silencing suppressor activity and cell death. Viruses 11 (11). 10.3390/v11110976 31652847 PMC6893708

[B28] Seegers S. L. Frasier C. Greene S. Nesmelova I. V. Grdzelishvili V. Z. (2020). Experimental evolution generates novel oncolytic vesicular stomatitis viruses with improved replication in virus-resistant pancreatic cancer cells. J. Virol. 94 (3). 10.1128/JVI.01643-19 31694943 PMC7000975

[B29] Shin D. H. Nguyen T. Ozpolat B. Lang F. Alonso M. Gomez-Manzano C. (2021). Current strategies to circumvent the antiviral immunity to optimize cancer virotherapy. J. Immunother. Cancer 9 (4), e002086. 10.1136/jitc-2020-002086 33795384 PMC8021759

[B30] Siegel R. L. Miller K. D. Fuchs H. E. Jemal A. (2022). Cancer statistics, 2022. CA Cancer J. Clin. 72 (1), 7–33. 10.3322/caac.21708 35020204

[B31] Stojdl D. F. Lichty B. D. tenOever B. R. Paterson J. M. Power A. T. Knowles S. (2003). VSV strains with defects in their ability to shutdown innate immunity are potent systemic anti-cancer agents. Cancer Cell 4 (4), 263–275. 10.1016/s1535-6108(03)00241-1 14585354

[B32] Sukumar M. Roychoudhuri R. Restifo N. P. (2015). Nutrient competition: a new axis of tumor immunosuppression. Cell 162 (6), 1206–1208. 10.1016/j.cell.2015.08.064 26359979 PMC6327313

[B33] Sung H. Ferlay J. Siegel R. L. Laversanne M. Soerjomataram I. Jemal A. (2021). Global cancer statistics 2020: GLOBOCAN estimates of incidence and mortality worldwide for 36 cancers in 185 countries. CA Cancer J. Clin. 71 (3), 209–249. 10.3322/caac.21660 33538338

[B34] Tesfay M. Z. Kirk A. C. Hadac E. M. Griesmann G. E. Federspiel M. J. Barber G. N. (2013). PEGylation of vesicular stomatitis virus extends virus persistence in blood circulation of passively immunized mice. J. Virol. 87 (7), 3752–3759. 10.1128/JVI.02832-12 23325695 PMC3624195

[B35] Tresilwised N. Pithayanukul P. Holm P. S. Schillinger U. Plank C. Mykhaylyk O. (2012). Effects of nanoparticle coatings on the activity of oncolytic adenovirus-magnetic nanoparticle complexes. Biomaterials 33 (1), 256–269. 10.1016/j.biomaterials.2011.09.028 21978891

[B36] Vander Heiden M. G. Cantley L. C. Thompson C. B. (2009). Understanding the Warburg effect: the metabolic requirements of cell proliferation. Science 324 (5930), 1029–1033. 10.1126/science.1160809 19460998 PMC2849637

[B37] Zainutdinov S. S. Kochneva G. V. Netesov S. V. Chumakov P. M. Matveeva O. V. (2019). Directed evolution as a tool for the selection of oncolytic RNA viruses with desired phenotypes. Oncolytic Virother 8, 9–26. 10.2147/OV.S176523 31372363 PMC6636189

