# Supplementary figures and images for: Tumor-directed evolution of VSVΔ51M produces novel viruses with enhanced antitumor efficacy
Source: Front Mol Biosci. 2026 May 8;13:1656006. doi: 10.3389/fmolb.2026.1656006 (PMC13202179; doi:10.3389/fmolb.2026.1656006)

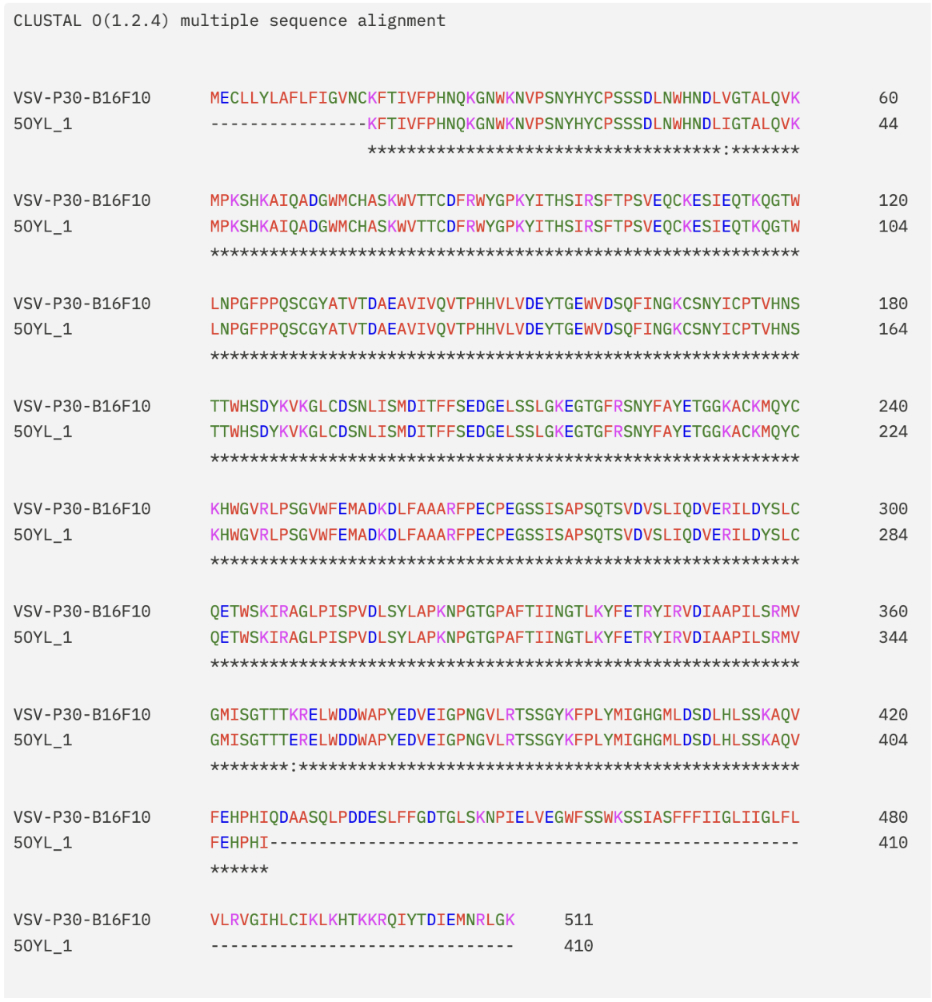

Supplement: Supplementary file 1 [file Image1.jpeg]
